# Supplementary material for: Multimorbidity, mortality, and HbA1c in type 2 diabetes: A cohort study with UK and Taiwanese cohorts
Source: PLoS Med. 2020 May 7;17(5):e1003094. doi: 10.1371/journal.pmed.1003094 (PMC7205223; doi:10.1371/journal.pmed.1003094)
Supplement: S1 Text — (DOCX) [file pmed.1003094.s001.docx]

**Text S1 – Study Protocol**

Original Study Protocol: UK Biobank/Taiwan NDCMP Diabetes Study

# Analysis Plan

## Scientific Research Title

Multimorbidity in Type 2 Diabetes: Associations With Mortality and HbA1c in Two Population Cohorts (UK and Taiwan)

## Research Question

- What is the relationship between multimorbidity (total; total of concordant conditions; total of discordant conditions) and all-cause mortality in people with type 2 diabetes?
- What is the relationship between multimorbidity (total; total of concordant conditions; total of discordant conditions) and HbA1c in people with type 2 diabetes?

### Study Design

**Type of Study:**

Longitudinal cohort study

## Methods

#### Inclusion Criteria

All participants part of the UK Biobank dataset with type 2 diabetes

All participants part of the Taiwan National Diabetes Care Management Program (NDCMP) dataset with type 2 diabetes

**Outcome variables:**

- All-cause mortality for patients with type 2 diabetes
- HbA1c in people with type 2 diabetes.

## Methods

**Sample size:**

UK biobank:

The sample size is based on the existing available data in the UK Biobank. This consists of approximately 500,000 people aged between 40-69 years. The data was collected throughout the UK from 2006-2010.

Those in the UK Biobank with a probable diagnosis of type 2 diabetes as defined by Eastwood et al (1) will be included in the analysis. N = 20,569

Taiwan NDCMP:

The sample size is based on the existing available data in the Taiwan NDCMP. This consists of approximately 60,000 people with diabetes recruited between 2001 and 2004, followed until 2011. Those with type 2 diabetes will be included in the analysis. N=59,657

## Statistical analysis

**Descriptive statistics**

Overall characteristics of the participants such as age, sex, socioeconomic status, smoking status, alcohol consumption, body mass index and diabetes duration will be summarised. The counts and proportions of chronic conditions concordant and discordant with diabetes will be summarised (see below tables for concordant and discordant chronic conditions). The list of chronic conditions utilised in this study is based on those described by Barnett et al (2) (see Table4).

**Statistical methods:**

**All-cause mortality**

A multivariable Cox proportional hazards model will be used to compare all-cause mortality between different categorical combinations of type 2 diabetes and the number of chronic conditions (total; total of concordant conditions; total of discordant conditions). Adjustments will be made for age, sex, Townsend score, smoking status, alcohol consumption, body mass index, physical activity, duration of diabetes and baseline HbA1c.

The hazard ratio, 95% confidence interval and p-values will be reported.

Multivariable Cox proportional hazards models will be fitted to all possible combinations of two conditions in addition to T2D to examine their association with all-cause mortality. We will present the top 20 combinations in terms of hazard ratios.

**HbA1c**

Multivariable mixed-effects linear regression will be used to examine the association between HbA1c (glycaemic control) and each of the multimorbidity counts (total; total of concordant conditions; total of discordant conditions) controlling for age, sex, Townsend score, smoking status, alcohol consumption, body mass index. physical activity and duration of diabetes. HbA1c will be treated as a continuous variable because the definition of what an optimal HbA1c is varies person to person and also country to country.

In all the regression models, we will treat the controlling factors as fixed effects.

The regression coefficient, 95% confidence interval and p-values will be reported.

**Results**

**Table 1. Characteristics of participants with type 2 diabetes**

| **Demographics** | **Total**  **N=** |
| --- | --- |
| Age, mean (SD) |  |
| Female, n(%) |  |
| Townsend score, n(%) |  |
| Category 1- least deprived (reference) |  |
| Category 2 |  |
| Category 3 |  |
| Category 4 |  |
| Category 5 – most deprived |  |
| Smoking status, n(%) |  |
| Current/previous |  |
| Never smoked |  |
| Alcohol Frequency, n(%) |  |
| Daily |  |
| 1-4 times/week |  |
| 1-3 times/month |  |
| BMI (kg/m^2^), n(%) |  |
| ≤18.5 |  |
| >18.5-25 |  |
| >25.0-30 |  |
| >30.0-35 |  |
| >35.0-40 |  |
| >40 |  |
| Physical Activity |  |
| None |  |
| Low |  |
| Medium |  |
| High |  |
| Duration of diabetes, median (IQR) |  |
| HbA1c (%), mean (SD) |  |
| Number of chronic conditions, n(%) |  |
| None |  |
| 1 |  |
| 2 |  |
| 3 |  |
| ≥4 |  |
| **Presence of chronic conditions concordant with type 2 diabetes, n(%)** |  |
| At least 1 chronic condition  concordant with diabetes |  |
| Hypertension |  |
| Coronary artery disease |  |
| Peripheral vascular disease |  |
| Chronic kidney disease |  |
| Stroke/TIA |  |
| Blindness and low vision  (from retinopathy) |  |
| Painful condition (from  neuropathy) |  |
| **Presence of chronic conditions discordant with type 2 diabetes, n(%)** |  |
| At least 1 chronic condition  discordant with diabetes |  |
| Heart failure |  |
| Depression |  |
| Painful condition (other than  from neuropathy) |  |
| Asthma |  |
| Dyspepsia |  |
| Thyroid disorders |  |
| Rheumatoid arthritis, other  Inflammatory polyarthropathies &  systematic connective tissue  disorders |  |
| COPD |  |
| Anxiety & other neurotic, stress  related & somatoform disorders |  |
| Irritable bowel syndrome |  |
| Cancer |  |
| Alcohol problems |  |
| Other psychoactive substance  misuse |  |
| Constipation |  |
| Diverticular disease of intestine |  |
| Atrial fibrillation |  |
| Heart failure |  |
| Prostate disorders |  |
| Glaucoma |  |
| Epilepsy (currently treated) |  |
| Dementia |  |
| Schizophrenia (and related non-  organic psychosis or bipolar  disorder) |  |
| Psoriasis or eczema |  |
| Inflammatory bowel disease |  |
| Migraine |  |
| Chronic sinusitis |  |
| Anorexia or bulimia |  |
| Bronchiectasis |  |
| Parkinson’s disease |  |
| Multiple sclerosis |  |
| Viral hepatitis |  |
| Chronic liver disease |  |
| *Osteoporosis |  |
| *Chronic fatigue syndrome |  |
| *Endometriosis |  |
| *Meniere’s disease |  |
| *Pernicious anaemia |  |
| *Polycystic ovary |  |

*Conditions not included in the Barnett paper, however are included because they have a prevalence of >2% in the dataset.

**Table 2. Regression results from Cox’s Proportional Hazards Model: Relationship between all-cause mortality and multimorbidity in participants with type 2 diabetes.**

| **Predictor variables** | **Hazard Ratios with 95% CI** | **P-value** |
| --- | --- | --- |
| **Age** |  |  |
| **Sex - male (against females as reference)** |  |  |
| **Townsend** |  |  |
| Category 1- least deprived (reference) |  |  |
| Category 2 |  |  |
| Category 3 |  |  |
| Category 4 |  |  |
| Category 5 – most deprived |  |  |
| **Smoking Status** |  |  |
| Never (reference) |  |  |
| Current/previous |  |  |
| **Alcohol Frequency** |  |  |
| Daily |  |  |
| 1-4 times/week (reference) |  |  |
| 1-3 times/month |  |  |
| **BMI** |  |  |
| <18.5 |  |  |
| 18.5-25 (reference) |  |  |
| 25.0-30 |  |  |
| >30 |  |  |
| **Duration of diabetes** |  |  |
| **Categories of diabetes and multimoribidties** |  |  |
| Diabetes present and no chronic conditions (reference) |  |  |
| Diabetes present and 1 chronic condition |  |  |
| Diabetes present and 2 chronic conditions |  |  |
| Diabetes present and 3 chronic conditions |  |  |
| Diabetes present and ≥4 chronic conditions |  |  |
| **Categories of diabetes and concordant conditions** |  |  |
| Diabetes present and no chronic conditions (reference) |  |  |
| Diabetes present and 1 concordant chronic condition |  |  |
| Diabetes present and 2 concordant chronic conditions |  |  |
| Diabetes present and 3 concordant chronic conditions |  |  |
| Diabetes present and ≥4 concordant chronic conditions |  |  |
| **Categories of diabetes and discordant conditons** |  |  |
| Diabetes present and no chronic conditions (reference) |  |  |
| Diabetes present and 1 discordant chronic condition |  |  |
| Diabetes present and 2 discordant chronic conditions |  |  |
| Diabetes present and 3 discordant chronic conditions |  |  |
| Diabetes present and ≥4 discordant chronic conditions |  |  |

**Table 3. Multivariable linear regression results: Relationship between multimorbidity and HbA1c in participants with type 2 diabetes.**

|  | **Unadjusted** | | | **Adjusted** | | |
| --- | --- | --- | --- | --- | --- | --- |
| **Predictor variables** | **β (SE)** | **95% CI** | **p** | **β (SE)** | **95% CI** | **p** |
| **Categories of diabetes and multimorbidities** |  |  |  |  |  |  |
| Diabetes present and no chronic conditions (reference) |  |  |  |  |  |  |
| Diabetes present and 1 chronic condition |  |  |  |  |  |  |
| Diabetes present and 2 chronic conditions |  |  |  |  |  |  |
| Diabetes present and 3 chronic conditions |  |  |  |  |  |  |
| Diabetes present and 4 or more chronic conditions |  |  |  |  |  |  |
| **Categories of diabetes and concordant conditions** |  |  |  |  |  |  |
| Diabetes present and no chronic conditions (reference) |  |  |  |  |  |  |
| Diabetes present and 1 concordant condition |  |  |  |  |  |  |
| Diabetes present and 2 concordant conditions |  |  |  |  |  |  |
| Diabetes present and 3 concordant conditions |  |  |  |  |  |  |
| Diabetes present and 4 or more concordant conditions |  |  |  |  |  |  |
| **Categories of diabetes and discordant conditions** |  |  |  |  |  |  |
| Diabetes present and no chronic conditions (reference) |  |  |  |  |  |  |
| Diabetes present and 1 discordant condition |  |  |  |  |  |  |
| Diabetes present and 2 discordant conditions |  |  |  |  |  |  |
| Diabetes present and 3 discordant conditions |  |  |  |  |  |  |
| Diabetes present and 4 or more discordant conditions |  |  |  |  |  |  |

**List of self-reported long term conditions considered for multimorbidity count**

Table 4. Definition of type 2 diabetes: Probable type 2 diabetes using algorithm developed by Eastwood et al (1)

| Long term conditions grouping | Conditions included |
| --- | --- |
| Concordant conditions | |
| 1. Hypertension | Hypertension  Essential hypertension |
| 1. Coronary heart Disease | Heart attack/Myocardial infarction  Angina |
| 1. Peripheral vascular disease | Peripheral vascular disease  Leg claudication/intermittent claudication |
| 1. Chronic kidney disease | Polycystic kidney  Diabetic nephropathy  Renal/kidney failure  Renal failure requiring dialysis  Renal failure not requiring dialysis  Kidney nephropathy  Immunoglobulin A (IgA) nephropathy |
| 1. Stroke/Transient Ischaemic Attack (TIA) | Stroke  TIA  Subarachnoid haemorrhage  Brain haemorrhage  Ischaemic stroke |
| 1. Diabetic retinopathy | Diabetic eye disease |
| 1. Diabetic neuropathy | Diabetic neuropathy/ulcers |
| 1. Atrial fibrillation | Atrial fibrillation |
| 1. Heart failure | Cardiomyopathy  Hypertrophic cardiomyopathy  Heart failure/pulmonary oedema |
| Discordant conditions | |
| 1. Depression | Depression  Postnatal depression |
| 1. Painful conditions | Back pain  Joint pain  Headaches (not migraine)  Sciatica  Plantar fasciitis  Carpal tunnel syndrome  Fibromyalgia  Arthritis  Shingles  Disc problem  Prolapsed disc/slipped disc  Spine arthritis/spondylitis  Ankylosing spondylitis  Back problem  Osteoarthritis  Gout  Cervical spondylosis  Trigeminal neuralgia  Disc degeneration  Trapped nerve/compressed nerve |
| 1. Asthma | Asthma |
| 1. Dyspepsia | Gastro-oesophageal reflux (GORD)/gastric reflux  Oesophagitis /Barrett's oesophagus  Gastric stomach ulcers  Gastric erosions/gastritis  Duodenal ulcer  Dyspepsia/indigestion  Hiatus hernia  Helicobacter pylori |
| 1. Thyroid disorders | Thyroid problem (not cancer)  Hyperthyroidism/thyrotoxicosis  Hypothyroidism/myxoedema  Grave’s disease  Thyroid goitre  Thyroiditis |
| 1. Rheumatoid arthritis and other connective tissue disorders | Myositis/myopathy  Systemic Lupus Erythematosus  Connective tissue disorder  Sjogrens syndrome/sicca syndrome  Dermatopolymyositis  Scleroderma/systemic sclerosis  Rheumatoid arthritis  Psoriatic arthropathy  Dermatomyositis  Polymyositis  Polymyalgia Rheumatica  Malabsorption/coeliac disease |
| 1. Irritable bowel syndrome | Irritable bowel syndrome |
| 1. Cancer | Lifetime diagnosis |
| 1. Alcohol problems | Alcohol dependency  Alcoholic liver disease/alcoholic cirrhosis |
| 1. Other psychoactive substance misuse | Opioid dependency  Other substance abuse/dependency |
| 1. Constipation | Constipation |
| 1. Diverticular disease | Diverticular disease  Diverticulitis |
| 1. Prostate disorders | Prostate problem (not cancer)  Enlarged prostate  Benign prostatic hypertrophy |
| 1. Glaucoma | Glaucoma |
| 1. Epilepsy | Epilepsy |
| 1. Dementia | Dementia  Alzheimer’s disease  Cognitive impairment |
| 1. Schizophrenia/bipolar disorder | Schizophrenia  Mania/  Bipolar disorder  Manic depression |
| 1. Psoriasis/eczema | Eczema  Dermatitis  Psoriasis |
| 1. Inflammatory bowel disease | Inflammatory Bowel Disease  Crohn’s disease  Ulcerative colitis |
| 1. Migraine | Migraine |
| 1. Chronic sinusitis | Chronic sinusitis |
| 1. Anorexia/bulimia | Anorexia  Bulimia  Other eating disorders |
| 1. Bronchiectasis | Bronchiectasis |
| 1. Parkinson’s disease | Parkinson’s disease |
| 1. Multiple sclerosis | Multiple sclerosis |
| 1. Viral hepatitis | Infective/viral hepatitis  Hepatitis B  Hepatitis C  Hepatitis D  Hepatitis E |
| 1. Chronic liver disease | Oesophageal varices  Non infective hepatitis  Liver failure/cirrhosis  Primary biliary cirrhosis |
| 1. Osteoporosis* | Osteoporosis |
| 1. Chronic fatigue syndrome* | Chronic fatigue syndrome |
| 1. Endometriosis* | Endometriosis |
| 1. Meniere’s disease* | Meniere’s disease |
| 1. Pernicious anaemia* | Pernicious anaemia |
| 1. Polycystic ovary* | Polycystic ovary |

**Total of 42 conditions (9 concordant & 33 discordant)**

*****Conditions are not included in the Barnett paper, however are included because they have a prevalence of >2% in the dataset.

### References

1. Eastwood SV, Mathur R, Atkinson M, Brophy S, Sudlow C, Flaig R, et al. Algorithms for the capture and adjudication of prevalent and incident diabetes in UK Biobank. PLOS One. 2016;11(9):1-18.

2. Barnett K, Mercer SW, Norbury M, Watt G, Wyke S, Guthrie B. Epidemiology of multimorbidity and implications for health care, research, and medical education: a cross-sectional study. The Lancet. 2012;380:37-43.
