# Supplementary material for: Multimorbidity, mortality, and HbA1c in type 2 diabetes: A cohort study with UK and Taiwanese cohorts
Source: PLoS Med. 2020 May 7;17(5):e1003094. doi: 10.1371/journal.pmed.1003094 (PMC7205223; doi:10.1371/journal.pmed.1003094)
Supplement: S1 Table — (DOCX) [file pmed.1003094.s003.docx]

**Table S1 – List of long-term conditions considered for multimorbidity count**

| Long term conditions grouping | Conditions included |
| --- | --- |
| Concordant conditions | |
| 1. Hypertension | Hypertension  Essential hypertension |
| 1. Coronary heart Disease | Heart attack/Myocardial infarction  Angina |
| 1. Peripheral vascular disease | Peripheral vascular disease  Leg claudication/intermittent claudication |
| 1. Chronic kidney disease | Polycystic kidney  Diabetic nephropathy  Renal/kidney failure  Renal failure requiring dialysis  Renal failure not requiring dialysis  Kidney nephropathy  Immunoglobulin A (IgA) nephropathy |
| 1. Stroke/Transient Ischaemic Attack (TIA) | Stroke  TIA  Subarachnoid haemorrhage  Brain haemorrhage  Ischaemic stroke |
| 1. Diabetic retinopathy | Diabetic eye disease |
| 1. Diabetic neuropathy | Diabetic neuropathy/ulcers |
| 1. Atrial fibrillation | Atrial fibrillation |
| 1. Heart failure | Cardiomyopathy  Hypertrophic cardiomyopathy  Heart failure/pulmonary oedema |
| Discordant conditions | |
| 1. Depression | Depression  Postnatal depression |
| 1. Painful conditions | Back pain  Joint pain  Headaches (not migraine)  Sciatica  Plantar fasciitis  Carpal tunnel syndrome  Fibromyalgia  Arthritis  Shingles  Disc problem  Prolapsed disc/slipped disc  Spine arthritis/spondylitis  Ankylosing spondylitis  Back problem  Osteoarthritis  Gout  Cervical spondylosis  Trigeminal neuralgia  Disc degeneration  Trapped nerve/compressed nerve |
| 1. Asthma | Asthma |
| 1. Dyspepsia | Gastro-oesophageal reflux (GORD)/gastric reflux  Oesophagitis /Barrett's oesophagus  Gastric stomach ulcers  Gastric erosions/gastritis  Duodenal ulcer  Dyspepsia/indigestion  Hiatus hernia  Helicobacter pylori |
| 1. Thyroid disorders | Thyroid problem (not cancer)  Hyperthyroidism/thyrotoxicosis  Hypothyroidism/myxoedema  Grave’s disease  Thyroid goitre  Thyroiditis |
| 1. Rheumatoid arthritis and other connective tissue disorders | Myositis/myopathy  Systemic Lupus Erythematosus  Connective tissue disorder  Sjogrens syndrome/sicca syndrome  Dermatopolymyositis  Scleroderma/systemic sclerosis  Rheumatoid arthritis  Psoriatic arthropathy  Dermatomyositis  Polymyositis  Polymyalgia Rheumatica  Malabsorption/coeliac disease |
| 1. Irritable bowel syndrome | Irritable bowel syndrome |
| 1. Cancer | Lifetime diagnosis |
| 1. Alcohol problems | Alcohol dependency  Alcoholic liver disease/alcoholic cirrhosis |
| 1. Other psychoactive substance misuse | Opioid dependency  Other substance abuse/dependency |
| 1. Constipation | Constipation |
| 1. Diverticular disease | Diverticular disease  Diverticulitis |
| 1. Prostate disorders | Prostate problem (not cancer)  Enlarged prostate  Benign prostatic hypertrophy |
| 1. Glaucoma | Glaucoma |
| 1. Epilepsy | Epilepsy |
| 1. Dementia | Dementia  Alzheimer’s disease  Cognitive impairment |
| 1. Schizophrenia/bipolar disorder | Schizophrenia  Mania/  Bipolar disorder  Manic depression |
| 1. Psoriasis/eczema | Eczema  Dermatitis  Psoriasis |
| 1. Inflammatory bowel disease | Inflammatory Bowel Disease  Crohn’s disease  Ulcerative colitis |
| 1. Migraine | Migraine |
| 1. Chronic sinusitis | Chronic sinusitis |
| 1. Anorexia/bulimia | Anorexia  Bulimia  Other eating disorders |
| 1. Bronchiectasis | Bronchiectasis |
| 1. Parkinson’s disease | Parkinson’s disease |
| 1. Multiple sclerosis | Multiple sclerosis |
| 1. Viral hepatitis | Infective/viral hepatitis  Hepatitis B  Hepatitis C  Hepatitis D  Hepatitis E |
| 1. Chronic liver disease | Oesophageal varices  Non infective hepatitis  Liver failure/cirrhosis  Primary biliary cirrhosis |
| 1. Osteoporosis | Osteoporosis |
| 1. Chronic fatigue syndrome | Chronic fatigue syndrome |
| 1. Endometriosis | Endometriosis |
| 1. Meniere’s disease | Meniere’s disease |
| 1. Pernicious anaemia | Pernicious anaemia |
| 1. Polycystic ovary | Polycystic ovary |
