# Supplementary material for: Multimorbidity, mortality, and HbA1c in type 2 diabetes: A cohort study with UK and Taiwanese cohorts
Source: PLoS Med. 2020 May 7;17(5):e1003094. doi: 10.1371/journal.pmed.1003094 (PMC7205223; doi:10.1371/journal.pmed.1003094)
Supplement: S2 Table — Relationship of multimorbidity total count with HbA1c. (DOCX) [file pmed.1003094.s004.docx]

**Table S2 – Sensitivity analysis: Relationship of multimorbidity total count with HbA1c in participants with type 2 diabetes using multivariable linear regression model in UK Biobank and Taiwan NDCMP**

|  | **UK Biobank** |  | **Taiwan NDCMP** |  |
| --- | --- | --- | --- | --- |
| **Predictor variables** | **Adjusted*** |  | **Adjusted**** |  |
| **Categories of diabetes and multimorbidity** | **Mean difference in HbA1c (95% CI)** | **P-value** | **Mean difference in HbA1c (95% CI)** | **P-value** |
| Diabetes present and no chronic conditions (reference) | ref |  | ref |  |
| Diabetes present and 1 chronic condition | -0.07 (-0.13, -0.01) | 0.027 | -0.44 (-0.49, -0.40) | <0.001 |
| Diabetes present and 2 chronic conditions | -0.13 (-0.18, -0.06) | <0.001 | -0.47 (-0.52, -0.42) | <0.001 |
| Diabetes present and 3 chronic conditions | -0.14 (-0.20, -0.07) | <0.001 | -0.44 (-0.50, -0.38) | <0.001 |
| Diabetes present and ≥4 chronic conditions | -0.21 (-0.28, -0.15) | <0.001 | -0.41 (-0.48, -0.35) | <0.001 |
| **Categories of diabetes and concordant conditions** |  |  |  |  |
| Diabetes present and no chronic conditions (reference) | Ref |  | ref |  |
| Diabetes present and 1 concordant chronic condition | -0.13 (-0.18, -0.07) | <0.001 | -0.22 (-0.26, -0.18) | <0.001 |
| Diabetes present and 2 concordant chronic conditions | -0.11 (-0.17, -0.04) | 0.001 | -0.20 (-0.25, -0.15) | <0.001 |
| Diabetes present and 3 concordant chronic conditions | -0.13 (-0.21, -0.04) | 0.003 | -0.14 (-0.23, -0.05) | 0.002 |
| Diabetes present and ≥4 concordant chronic conditions | -0.06 (-0.19, 0.07) | 0.366 | -0.05 (-0.10, -0.21) | 0.509 |
| **Categories of diabetes and discordant conditions** |  |  |  |  |
| Diabetes present and no chronic conditions (reference) | ref |  | ref |  |
| Diabetes present and 1 discordant chronic condition | -0.12 (-0.17, -0.06) | <0.001 | -0.20 (-0.24, -0.16) | <0.001 |
| Diabetes present and 2 discordant chronic conditions | -0.15 (-0.21, -0.08) | <0.001 | -0.17 (-0.22, -0.12) | <0.001 |
| Diabetes present and 3 discordant chronic conditions | -0.22 (-0.29, -0.14) | <0.001 | -0.19 (-0.26, -0.12) | <0.001 |
| Diabetes present and ≥4 discordant chronic conditions | -0.25 (-0.33, -0.17) | <0.001 | -0.04 (-0.13, -0.06) | 0.411 |

* Adjusting for age, gender, BMI, smoking status, alcohol consumption, socioeconomic status, baseline HbA1c, duration of diabetes, use of oral anti-diabetes drugs and use of corticosteroids, and physical activity

****** Adjusting for age, gender, BMI, smoking status, alcohol consumption, socioeconomic status, baseline HbA1c, duration of diabetes, use of oral anti-diabetes drugs, use of corticosteroids, and number of outpatient visits
