# Supplementary material for: Multimorbidity, mortality, and HbA1c in type 2 diabetes: A cohort study with UK and Taiwanese cohorts
Source: PLoS Med. 2020 May 7;17(5):e1003094. doi: 10.1371/journal.pmed.1003094 (PMC7205223; doi:10.1371/journal.pmed.1003094)
Supplement: S3 Table — Relationship of multimorbidity total count with all-cause mortality. (DOCX) [file pmed.1003094.s005.docx]

**Table S3 – Sensitivity analysis: Relationship between multimorbidity total count and all-cause mortality in participants with type 2 diabetes using multivariable Cox’s Proportional Hazards model in UK Biobank and Taiwan NDCMP**

|  | **UK Biobank** | | **Taiwan NDCMP** | |
| --- | --- | --- | --- | --- |
| **Predictor variables** | **Adjusted*** | | **Adjusted**** | |
| **Categories of diabetes and multimorbidity** | **HRs (95% CI)** | **P-value** | **HRs (95% CI)** | **P-value** |
| Diabetes present and no chronic conditions (reference) | 1 |  | 1 |  |
| Diabetes present and 1 chronic condition | 1.14 (0.89, 1.48) | <0.001 | 1.19 (1.11, 1.27) | <0.001 |
| Diabetes present and 2 chronic conditions | 1.69 (1.32, 2.16) | <0.001 | 1.43 (1.33, 1.52) | <0.001 |
| Diabetes present and 3 chronic conditions | 2.03 (1.58, 2.61) | <0.001 | 1.84 (1.72, 1.98) | <0.001 |
| Diabetes present and ≥4 chronic conditions | 2.91 (2.28, 3.71) | <0.001 | 2.60 (2.42, 2.80) | <0.001 |
| **Categories of diabetes and concordant conditions** |  |  |  |  |
| Diabetes present and no chronic conditions (reference) | 1 |  | 1 |  |
| Diabetes present and 1 concordant chronic condition | 1.52 (1.19, 1.96) | <0.001 | 1.22 (1.16, 1.27) | <0.001 |
| Diabetes present and 2 concordant chronic conditions | 2.28 (1.76, 2.96) | <0.001 | 1.60 (1.51, 1.69) | <0.001 |
| Diabetes present and 3 concordant chronic conditions | 3.79 (2.87, 5.01) | <0.001 | 2.38 (2.21, 2.56) | <0.001 |
| Diabetes present and ≥4 concordant chronic conditions | 5.26 (3.83, 7.21) | <0.001 | 3.19 (2.86, 3.55) | <0.001 |
| **Categories of diabetes and discordant conditions** |  |  |  |  |
| Diabetes present and no chronic conditions (reference) | 1 |  | 1 |  |
| Diabetes present and 1 discordant chronic condition | 1.83 (1.42, 2.36) | <0.001 | 1.15 (1.10, 1.20) | <0.001 |
| Diabetes present and 2 discordant chronic conditions | 1.99 (1.53, 2.60) | <0.001 | 1.32 (1.25, 1.39) | <0.001 |
| Diabetes present and 3 discordant chronic conditions | 2.44 (1.85, 3.23) | <0.001 | 1.63 (1.53, 1.74) | <0.001 |
| Diabetes present and ≥4 discordant chronic conditions | 3.22 (2.43, 4.26) | <0.001 | 2.06 (1.91, 2.23) | <0.001 |

* Adjusting for age, gender, BMI, smoking status, alcohol consumption, socioeconomic status, baseline HbA1c, duration of diabetes, use of oral anti-diabetes drugs and use of corticosteroids, and physical activity

****** Adjusting for age, gender, BMI, smoking status, alcohol consumption, socioeconomic status, baseline HbA1c, duration of diabetes, use of oral anti-diabetes drugs, use of corticosteroids, and number of outpatient visits
