# Supplementary material for: Multimorbidity, mortality, and HbA1c in type 2 diabetes: A cohort study with UK and Taiwanese cohorts
Source: PLoS Med. 2020 May 7;17(5):e1003094. doi: 10.1371/journal.pmed.1003094 (PMC7205223; doi:10.1371/journal.pmed.1003094)
Supplement: S4 Table — Hospital-verified data. (DOCX) [file pmed.1003094.s006.docx]

**Table S4 – Sensitivity analysis using only hospital verified data for identifying chronic conditions**

**Prevalence of individual multimorbid conditions in participants with type 2 diabetes in the UK Biobank using hospital data only, and combined self-report and hospital data**

|  | **Using only hospital data** | **Using both self-report and hospital data**  **(Table 2 from manuscript)** |
| --- | --- | --- |
| **Presence of chronic conditions concordant with type 2 diabetes, n (%)** | **UK Biobank**  **(N = 20,569)** | **UK Biobank**  **(N = 20,569)** |
| Hypertension | 5632 (27.4) | 14,187 (69.0) |
| Coronary heart disease | 2918 (14.2) | 3,773 (18.3) |
| Peripheral vascular disease | 429 (2.1) | 488 (2.4) |
| Chronic kidney disease | 244 (1.2) | 323 (1.6) |
| Stroke/TIA | 434 (2.1) | 1,024 (5.0) |
| Diabetic retinopathy | 2,174 (10.6) | 2,174 (10.6) |
| Diabetic neuropathy | 74 (0.4) | 74 (0.4) |
| Atrial fibrillation | 641 (3.1) | 641 (3.1) |
| Heart failure | 389 (1.9) | 426 (2.1) |
| **Presence of chronic conditions discordant with type 2 diabetes, n (%)** | **UK Biobank**  **(N = 20,569)** | **UK Biobank**  **(N = 20,569)** |
| Depression | 419 (2.0) | 1,643 (8.0) |
| Painful conditions (excluding diabetic neuropathy) | 3140 (15.3) | 6,250 (30.4) |
| Asthma | 1162 (5.6) | 2,959 (14.4) |
| Dyspepsia | 2528 (12.3) | 3,815 (18.5) |
| Thyroid disorders | 627 (3.0) | 1,688 (8.2) |
| Rheumatoid arthritis and other connective tissue disorders | 275 (1.3) | 618 (3.0) |
| COPD | 397 (1.9) | 841 (4.1) |
| Anxiety | 181 (0.9) | 509 (2.5) |
| Irritable bowel syndrome | 185 (0.9) | 500 (2.4) |
| Cancer | 1477 (7.2) | 2,110 (10.3) |
| Alcohol problems | 402 (2.0) | 427 (2.1) |
| Other psychoactive substance misuse | 10 (0.0) | 13 (0.1) |
| Constipation | 275 (1.3) | 288 (1.4) |
| Diverticular disease | 895 (4.4) | 1,056 (5.1) |
| Prostate disorders | 516 (2.5) | 890 (4.3) |
| Glaucoma | 152 (0.7) | 458 (2.2) |
| Epilepsy | 132 (0.6) | 211 (1.0) |
| Dementia | 8 (0.0) | 10 (0.0) |
| Schizophrenia/bipolar disorder | 76 (0.4) | 187 (0.9) |
| Psoriasis/eczema | 170 (0.8) | 792 (3.9) |
| Inflammatory bowel disease | 871 (4.2) | 924 (4.5) |
| Migraine | 57 (0.3) | 306 (1.5) |
| Chronic sinusitis | 87 (0.4) | 176 (0.9) |
| Anorexia/bulimia | 0 (0.0) | 2 (0.0) |
| Bronchiectasis | 37 (0.2) | 56 (0.3) |
| Parkinson’s disease | 23 (0.2) | 42 (0.2) |
| Multiple sclerosis | 49 (0.2) | 71 (0.3) |
| Viral hepatitis | 28 (0.1) | 56 (0.3) |
| Chronic liver disease | 302 (1.5) | 326 (1.6) |
| Osteoporosis | 141 (0.7) | 340 (1.7) |
| Chronic fatigue syndrome | 10 (0.0) | 71 (0.3) |
| Endometriosis | 84 (0.4) | 162 (0.8) |
| Meniere’s disease | 13 (0.1) | 52 (0.3) |
| Pernicious anaemia | 20 (0.1) | 134 (0.7) |
| Polycystic ovary | 10 (0.0) | 31 (0.2) |

Abbreviations: T2D, type 2 diabetes; TIA, transient ischaemic attack; COPD, chronic obstructive pulmonary disease

**Relationship between multimorbidity total count and HbA1c in participants with type 2 diabetes using multivariable linear regression model in UK Biobank**

|  | **Using only hospital data** | | | | **Using both self-report and hospital data**  **(Table 3 from manuscript)** | | | |
| --- | --- | --- | --- | --- | --- | --- | --- | --- |
|  | **Unadjusted** | | **Adjusted*** | | **Unadjusted** | | **Adjusted*** |  |
| **Categories of diabetes and multimorbidity** | **Mean difference in HbA1c (95% CI)** | **P-value** | **Mean difference in HbA1c (95% CI)** | **P-value** | **Mean difference in HbA1c (95% CI)** | **P-value** | **Mean difference in HbA1c (95% CI)** | **P-value** |
| Diabetes only (reference) | ref |  | ref |  | ref |  | ref |  |
| Diabetes plus 1 chronic condition | -0.01 (-0.05, 0.03) | 0.687 | -0.02 (-0.13, -0.01) | 0.448 | -0.07 (-0.14, -0.01) | 0.024 | -0.07 (-0.13, -0.01) | 0.031 |
| Diabetes plus 2 chronic conditions | -0.03 (-0.08, 0.02) | 0.208 | -0.03 (-0.18, -0.06) | 0.270 | -0.13 (-0.19, -0.06) | <0.001 | -0.12 (-0.18, -0.06) | <0.001 |
| Diabetes plus 3 chronic conditions | -0.07 (-0.13, -0.01) | 0.025 | -0.08 (-0.19, -0.06) | 0.006 | -0.11 (-0.17, -0.04) | 0.002 | -0.13 (-0.19, -0.06) | <0.001 |
| Diabetes plus ≥4 chronic conditions | -0.09 (-0.15, -0.03) | 0.002 | -0.13 (-0.26, -0.14) | <0.001 | -0.16 (-0.23, -0.10) | <0.001 | -0.20 (-0.26, -0.14) | <0.001 |

* Adjusting for age, gender, BMI, smoking status, alcohol consumption, socioeconomic status, baseline HbA1c, duration of diabetes, use of oral anti-diabetes drugs and use of corticosteroids

**Relationship between multimorbidity total count and all-cause mortality in participants with type 2 diabetes using multivariable Cox’s Proportional Hazards model in UK Biobank**

|  | **Using only hospital data** | | | | **Using both self-report and hospital data**  **(Table 4 from manuscript)** | | | |
| --- | --- | --- | --- | --- | --- | --- | --- | --- |
|  | **Unadjusted** | | **Adjusted*** | | **Unadjusted** | | **Adjusted*** | |
| **Categories of diabetes and multimorbidity** | **HRs (95% CI)** | **P-value** | **HRs (95% CI)** | **P-value** | **HRs (95% CI)** | **P-value** | **HRs (95% CI)** | **P-value** |
| Diabetes only (reference) | 1 |  | 1 |  | 1 |  | 1 |  |
| Diabetes plus 1 chronic condition | 1.48 (1.30, 1.69) | <0.001 | 1.33 (1.15, 1.52) | <0.001 | 1.33 (1.04, 1.71) | 0.024 | 1.20 (0.91, 1.56) | <0.001 |
| Diabetes plus 2 chronic conditions | 1.89 (1.64, 2.16) | <0.001 | 1.58 (1.36, 1.83) | <0.001 | 2.04 (1.61, 2.60) | <0.001 | 1.75 (1.35, 2.27) | <0.001 |
| Diabetes plus 3 chronic conditions | 2.71 (2.35, 3.14) | <0.001 | 2.10 (1.79, 2.46) | <0.001 | 2.73 (2.14, 3.48) | <0.001 | 2.17 (1.67, 2.81) | <0.001 |
| Diabetes plus ≥4 chronic conditions | 4.20 (3.70, 4.77) | <0.001 | 3.26 (2.83, 3.75) | <0.001 | 4.14 (3.28, 5.22) | <0.001 | 3.14 (2.43, 4.03) | <0.001 |

* Adjusting for age, gender, BMI, smoking status, alcohol consumption, socioeconomic status, baseline HbA1c, duration of diabetes, use of oral anti-diabetes drugs and use of corticosteroids
