# Supplementary material for: Multimorbidity, mortality, and HbA1c in type 2 diabetes: A cohort study with UK and Taiwanese cohorts
Source: PLoS Med. 2020 May 7;17(5):e1003094. doi: 10.1371/journal.pmed.1003094 (PMC7205223; doi:10.1371/journal.pmed.1003094)
Supplement: S5 Table — (DOCX) [file pmed.1003094.s007.docx]

**Table S5 – Spline plots of multimorbidity condition count and mortality in a) the UK Biobank and b) the Taiwan NDCMP**

| **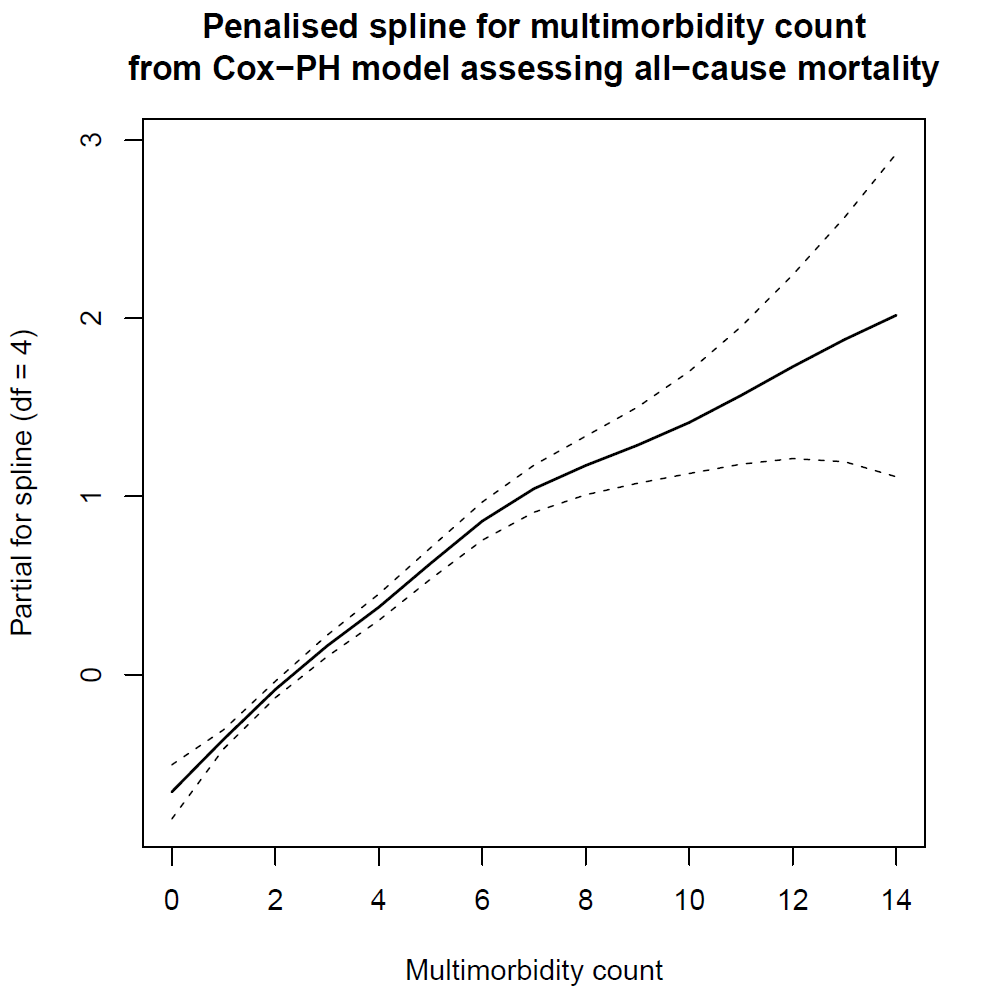**  **a** |
| --- |
| **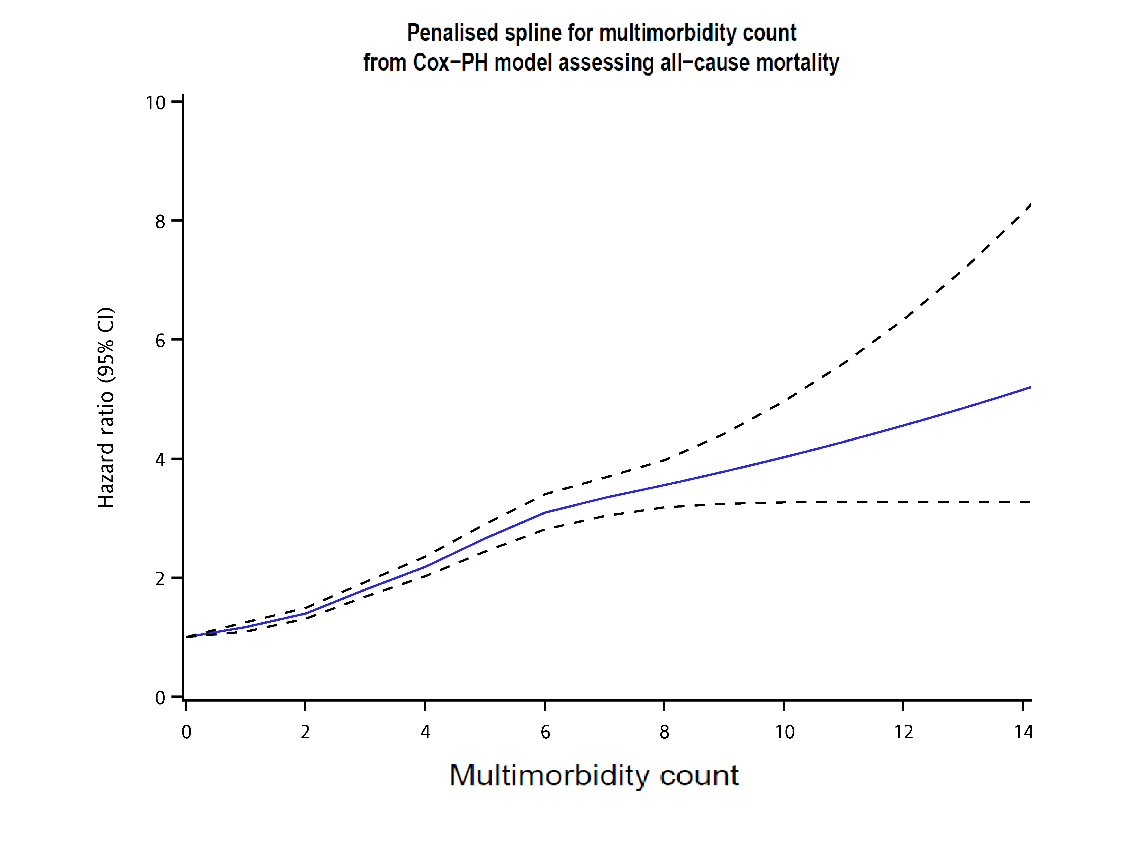 b** |
