# Supplementary material for: Multimorbidity, mortality, and HbA1c in type 2 diabetes: A cohort study with UK and Taiwanese cohorts
Source: PLoS Med. 2020 May 7;17(5):e1003094. doi: 10.1371/journal.pmed.1003094 (PMC7205223; doi:10.1371/journal.pmed.1003094)
Supplement: Attachment — Submitted filename: Letter Response to Reviewers.docx [file pmed.1003094.s008.docx]

30^th^ January 2020

Dr Clare Stone

PLOS Medicine

Dear Dr Stone,

**RE: PMEDICINE-D-19-03306 – Multimorbidity in Type 2 Diabetes: Associations With Mortality and HbA1c in Two Population Cohorts (UK and Taiwan)**

Thank you for the opportunity to re-submit this paper for consideration of publication in PLOS Medicine.

I would like to thank the reviewers for their helpful comments and constructive feedback, which has improved our paper. I have responded to each comment, with changes made to the paper indicated in the Detailed Response to Reviewers below.

I look forward to hearing the outcome of the review of our paper.

Best regards,

Jason Chiang

On behalf of the authors

**Detailed Response to Reviewers**

| **Comment** | | **Response** |
| --- | --- | --- |
| ***Requests from the Editors:*** | | |
| 1 | The abstract is written is a slightly ‘listy’ way with headings of outcomes etc. Please remove and write in a more traditional style and avoid short statements such as ‘Baseline HbA1c; all-cause mortality’;  as the final sentence of the ‘Methods and findings’ section please add a sentence or 2 on the limitations of the study;  please add p values where 95%Cis are given;  please add some baseline demographic information on the participants in the cohorts | Thank you for the feedback. We rectified this and have written the abstract in a more traditional style with full sentences. (See page 2)  We have also added on the limitations of the study at the end of “Methods and Findings.”  We have added the following sentence to page 2 lines 58-59 “One limitation to note is that we were unable to model for changes in multimorbidity during our study period.”  P-values have been added where 95%Cis are given and brief baseline demographics on participants have also been added.  We have added the following sentence on page 2 “Mean (SD) age for the UK Biobank and Taiwan NDCMP were 60.2 (6.8) years and 60.8 (11.3) years, respectively.” |
| 2 | At this stage, we ask that you include a short, non-technical Author Summary of your research to make findings accessible to a wide audience that includes both scientists and non-scientists. The Author Summary should immediately follow the Abstract in your revised manuscript. This text is subject to editorial change and should be distinct from the scientific abstract. | We have added the Author Summary section immediately following the abstract of our revised manuscript. It can be found on pages 4-5. |
| 3 | Square brackets for refs in main text – please place these before the punctuation. And when occurring mid-sentence please insert a space before the open bracket. | All the references have been corrected throughout the manuscript. |
| 4 | The use of MM throughout – as this probably generates lots of returns in PubMed searches please avoid using MM. I also think co-morbidity is a more commonly used phrase? So in any case remove ‘MM’ throughout, please and consider instead co-morbidity (spelt out in full and not as CM). Further to this, in both the abstract and main text on first mention, can you please list what these other conditions are or at least the major ones. | Thanks for the comment. We have removed the use of MM and now use “multimorbidity” throughout, increasing our manuscripts readability. |
| 5 | Please avoid use of quote marks (“”) for emphasis or quoting other papers. | We have removed all quotation marks. (See page 6) |
| 6 | Line 133 – can you clarify what light ‘do-it-yourself (DIY)’ activity only in the last 4week means? Are you defining exercise for this category as only doing DIY? It reads oddly to say exercise is DIY. | Thank you for the comment. We have added the following text on page 8 lines 202-205 to clarify how we categorised physical activity and defined DIY exercises.  “Physical activity was self-reported based on responses from the UK Biobank physical activity questionnaire. We categorised the responses into: none (no physical activity in the last 4weeks); low (light ‘do-it-yourself (DIY)’ activity [eg, pruning, watering the lawn] only in the last 4weeks); medium (heavy DIY [eg, weeding, lawn mowing, carpentry and digging] and/or walking for pleasure and/or other exercises in the last 4 weeks); or high (strenuous sports in the last 4 weeks).” |
| 7 | Table 3, please remove ‘present’ after diabetes and write as ‘diabetes plus….’ (also for Table 4 and any other similar occurences, please). | Thanks for the suggestion, this makes the tables much cleaner. We have changed the wording in Tables 3 and 4 as per the Editor’s suggestion. |
| 8 | Fig 1 – add a time scale (dates) to the X axis in all panels | Thank you for the comment. We were not able to add dates to the X axis in all panels of Fig 1 because the recruitment dates for the UK Biobank and the Taiwan NDCMP varies for different people. Participants in the UK Biobank were recruited between 2006 and 2010, and the participants in the Taiwan NDCMP were recruited between 2001 and 2004. However, we have relabelled “0” to “Recruitment” on the X axis. |
| 9 | Main text – please add p values with Cis | We have added p-values to all the CIs in the manuscript. |
| 10 | As Ref 3 also mentions, please avoid causal language as this is not a trial | Thank you for the suggestion. We have reworded all causal language statements throughout the manuscript. |
| 11 | STROBE checklist – thank you for supplying – please also add the paragraph numbers to the sections. | We have added paragraph numbers to the STROBE checklist. |
| ***Reviewer 1***  I confine my remarks to statistical aspects of this paper. I have a couple general questions: | | |
| 1 | Why treat count independent variables as if they were nominal? Even with the "4 or more" category, it is at least ordinal. Ordinal IVs are a bit tricky, but one method is optimal scaling. In SAS this is avaiable in PROC TRANSREG. In R, there is the optiscale package. But, since you have the actual count, you could treat it as continuous and maybe use a spline to see if there are nonlinearities. | Thank you for this very thoughtful comment. We have had extensive discussions amongst the author group on the approach we should take in presenting our multimorbidity condition counts. We have made the decision to treat the multimorbidity condition counts as categorical variables. Our author group, which includes academic clinicians, believes that presenting multimorbidity condition count as a categorical variable and showing the associations between each of the categories and our outcomes of interest (HbA1c and all-cause mortality) makes the results easier to interpret clinically and may be easier to translate into real world clinical practice. Furthermore, treating multimorbidity condition count in this way have been used in a number of recently published papers exploring multimorbidity (1-3). Nevertheless, we have taken your suggestion and used spline to see whether there are nonlinearities when treating multimorbidity condition count as continuous (See Table S5 in the appendix). The relationship between multimorbidity condition count and mortality seem to be linear until a count of 7 in both the UK Biobank and the Taiwan NDCMP. We also used fractional polynomials to model non-linear relationships between multimorbidity condition count and mortality, which did not improve the fit of the models.  Ultimately, we have made the decision to treat multimorbidity condition count as categorical given that the results easier to interpret clinically, allow comparison with other recently published studies, and that treating count as continuous or categorical provide similar results.  1. Jani BD, Hanlon P, Nicholl BI, McQueenie R, Gallacher KI, Lee D, et al. Relationship between multimorbidity, demographic factors and mortality: findings from the UK Biobank cohort. BMC Med. 2019;17(1):74.  2. Jani BD, Nicholl BI, McQueenie R, Connelly DT, Hanlon P, Gallacher KI, et al. Multimorbidity and co-morbidity in atrial fibrillation and effects on survival: findings from UK Biobank cohort. Europace 2018;20(FI_3):f329-f36.  3. Gallacher KI, McQueenie R, Nicholl B, Jani BD, Lee D, Mair FS. Risk factors and mortality associated with multimorbidity in people with stroke or transient ischaemic attack: a study of 8,751 UK Biobank participants. Journal of comorbidity. 2018;8(1):1-8. |
| 2 | Although it isn't necessary, I think it would be very interesting to see if the effects of comorbities are additive or not, at least for the more common examples. That is, is the increased risk of death for people with T2D and CHD equal to the increase from T2D PLUS the increase from CHD? Or is it more? Or less? (This would require a normative sample .... it might even be a different paper). | Thank you for this thoughtful suggestion. We agree that it would be interesting to see whether the effects of additional conditions are additive. However, we also agree with the reviewer that this is a separate issue for a different paper. Therefore, we have made the decision not to explore this given our manuscript is already a comprehensive one with numerous outcomes and novel findings. Furthermore, this would involve extensive additional analyses, adding to the length of the paper. |
| 3 | Line 120 - why group 4 or more? It's OK for tables, but not a great idea for models (see above) | Thank you for the suggestion. We have grouped “4 or more” so that we would have similar numbers of participants in each of the groups. |
| 4 | Line 125-126 Don't categorize BMI. Categorizing continuous variables is almost always a mistake.  Similar issues for amount of premium in Taiwan | Thanks for the suggestion. We are now treating BMI for both UK Biobank and Taiwan NDCMP, and amount of premium for Taiwan NDCMP as continuous variables. We have updated the texts in the methods section (page 8 lines 192), the numbers in Table 1 (pages 10-11), and also made the changes in all of our models. |
| 5 | Line 130 - why group current and former smokers? | Thank you for the comment. We have changed the way we categorised smoking status for the UK Biobank so it is now consistent with the Taiwan categories. The categories are now: yes (current smokers); and no. We have updated the texts in the methods section (page 8 line 199), numbers in Table 1 (page 10-11), and also made the changes in all of our models. |
| 6 | Line 131-132 - if this is how the data were collected on alcohol, well ... then there isn't much you can do. But surely risk increases a lot at the high end (people who binge driink or drink many drinks per day) | Unfortunately, we are not able to change this as this was how data on alcohol consumption was collected for the UK Biobank. |
| 7 | Line 138-139 - same sort of question as above. | Unfortunately, we are not able to change this as this was how data on alcohol consumption and smoking status were collected for the Taiwan NDCMP. |
| 8 | Line 143 - maybe I am misunderstanding something, but how is baseline HbA1c an outcome? | Thank you for this helpful comment. We have added the following text in the methods section on page 8 lines 213-215 to clarify this.  “We explored the cross-sectional association between multimorbidity counts and the most recent HbA1c measure collected at time of recruitment. In contrast, the association between multimorbidity counts and all-cause mortality explored was longitudinal.” |
| 9 | Table 1, 2 :  Give median and IQR for BMI  The order of alcohol in table 1 is odd  For the IQR for duration, give it as 2 numbers (e.g 2 to 10). This is much more informative | Thank you for the suggestion. We have rectified this and the numbers are now much more informative. (See pages 10-11) |
| 10 | Table 3 strikes me as very weird. However, given that the mean HbA1c was 6.8, are any of these differences substantively meaningful? (I know some are significant .... but is 6.75 really different from 6.85 in any important way? | Thank you for this thoughtful comment. We agree that although our results show that there are statistically significant associations between increasing multimorbidity count and lower HbA1c, the degree of differences observed in HbA1c are not likely to be clinically significant. We have added the following text to our discussion section (see page 30 lines 391-402) to expand on this point.  “Although our results indicate associations between increasing multimorbidity and slightly lower HbA1c, a major caveat is that the degree of difference observed in HbA1c (ranging from -0.07% to -0.82%) seen in our results is not likely to be clinically significant despite being statistically significant. There have been discussions around what degree of reduction in HbA1c could be seen as clinically significant however, it would be patient dependent [22]. Not all HbA1c improvements are equal in regard to clinical benefit, for example a reduction of 1% in HbA1c offers different benefit if the improvement was from 12% to 11% compared to 7% to 6%. Furthermore, it is generally regarded that it is easier for patients with poorer glycaemic control to reduce their HbA1c (for example, from 10% to 9%) than for patients with relatively good glycaemic control. While UK Biobank cohort, represents a relatively healthy population with good glycaemic control where the mean (SD) HbA1c was 6.8 (1.2)%, it is important to note that the results were similar in the Taiwan N.DCMP cohort, which is a population cohort where the mean (SD) HbA1c was 8.2 (2.0)%.” |
| 11 | Figures - I would label the curves on the right hand margin with 0, 1, 2, 3, 4+ That seems more readable. | Thank you for the suggestion. We have re-labelled all of the curves with 0, 1, 2, 3, 4+ on the right hand margins. |
| ***Reviewer 2***  This paper examines the mortality and HbA1c in people with type 2 diabetes and multimorbidity. This is a highly relevant research question given the rapidly raising number of people with diabetes and multimorbidity, and the novelty of the topic.  The strengths of the study include two large cohorts from two countries with two different healthcare systems and different ethnicities, combined with extended follow-up durations. The authors found that increasing total MM and discordant condition counts were associated with slightly lower HbA1c and significant associations between increasing MM and risk of mortality, which was consistent for total count of MM, as well as counts of, concordant and discordant conditions.  The manuscript is clearly and succinctly written and addresses a novel aspect of a subject of increasing interest and importance, and of notable clinical relevance. Nonetheless, I would say the results are less surprising, I have the following concerns: | | |
| 1 | The conditions for the UK Biobank was ascertained either by self-reported or linked hospital data. This means that other conditions were assessed imprecisely and a large proportion of diabetes cases were missed as the ascertainment method did not include undiagnosed diabetes. It is unclear how this affects on study conclusion. I suggest the author re-run the models by using verified hospital cases to see whether the results were the same as combing both self-reported and hospital verified data. | Thank you for this thoughtful comment. We have added the following text to our methods section (page 7 lines 164-166)addressing the validity of identifying those with type 2 diabetes in the UK Biobank using the Eastwood algorithm which has been validated externally with primary care and secondary care hospital data.  “We identified 20,569 people with T2D using a published algorithm developed by Eastwood et al. [13], which has been validated externally using primary care and secondary care hospital data linked to the UK Biobank [13].”  Based on your suggestion we have also re-examined the prevalence of each of the conditions included in our multimorbidity count only using hospital data and re-ran the HbA1c and all-cause mortality models only using hospital data (see Table S4 in the appendix). We then compared the results with the models in our manuscript where we used a combination of self-report and hospital data. The prevalence of conditions only using hospital data was lower than using a combination of both self-report and hospital data and thus may under-identify the conditions participants may be living with. However, when we compared the results of the HbA1c and all-cause mortality results using only hospital data, the results were similar to that of the results using the combined self-report and hospital data. We have added the following text to the sensitivity analysis section of our methods section (see page 9 lines 255) to outline this additional analysis only using hospital data.  “For the multivariable linear regression models on HbA1c and multivariable Cox proportional hazards models on mortality for the UK Biobank we re-ran the models only using HES data to identify multimorbidity.”  We have also added the following text to comment on the similar results between models using hospital data only vs combined self-report and hospital data to the respective HbA1c (see page 13 lines 290-292) and all-cause mortality sections (see page 19 lines 319-321).  “When we used only the HES data to identify multimorbidity in the UK Biobank, the results for associations between multimorbidity and HbA1c were similar to our main analysis (Table S4).” (see page 13 lines 290-292)  “When we used only the HES data to identify multimorbidity in the UK Biobank, the results for associations between multimorbidity and all-cause mortality were similar to our main analysis (Table S4).” (see page 19 lines 319-321) |
| 2 | Although the authors adjusted the duration of the diabetes, what about the duration of other conditions? This is very important for some fatal conditions, for example, cancer, stork and TIA, which may take into account the competing risk, that is, the higher mortality risk in patients with diabetes and these conditions was because these conditions were the first occurring ones, and diabetes was a secondary one. | Thank you for the comment. We agree that the duration of other conditions is also important to consider, particularly for fatal conditions including stroke, TIA and cancer. Unfortunately, the Taiwan NDCMP dataset does not have the data to explore the duration of conditions and we therefore do not have the capacity to explore the duration of conditions in addition to diabetes. We have added the following text in our strengths and limitations section on page 32 lines 454-458 to address this limitation.  “However, these data were only available at baseline and we were unable to model for changes in multimorbidity over our study period. Therefore, a limitation of our study is that we were unable to consider the temporality and duration of the conditions in addition to diabetes, which is important for serious conditions such as stroke, TIA and cancer.” |
| 3 | Although there was some evidence to support the classification of these conditions into concordant and discordant, it's still controversial for some, for example, depression and anxiety, many studies suggest that these mental disorders and diabetes shared some common pathways. The discussion of this point and the potential resulting limitations should be expanded upon in the "Strengths and limitations" section of the Discussion. | Thank you for the suggestion. We have added the following text and an additional reference to our strengths and limitations section on page 32 lines 467-471 to expand on the classification of mental health conditions and it could result in limitations.  “Although we have classified mental health conditions including depression and anxiety as discordant conditions, there are still debates on whether this is appropriate. Studies have shown that depression and anxiety may share biological and behavioural mechanisms [32], which could mean that our classification of these conditions could lead to underestimating the associations between concordant conditions and our outcomes.”  32. Holt RI, de Groot M, Golden SH. Diabetes and depression. Curr Diab Rep. 2014;14(6):491. Epub 2014/04/20. doi: 10.1007/s11892-014-0491-3. PubMed PMID: 24743941; PubMed Central PMCID: PMCPMC4476048. |
| 4 | The prevalence of concordant and discordant conditions in the UK Biobank were 76.1% and 66.9% separately, there is a huge overlap between the two condition groups. However, it seems the authors did not explain what happened and how to deal with this overlap. If one give person has suffered from both concordant and discordant conditions, can the author figure out the effect on the HbA1c and mortality from which condition group? Furthermore, there are also overlaps among the included discordant conditions, for example, it's obvious that many people with alcohol problems would develop chronic liver diseases, depression and other conditions. It means the effects of alcohol problems may from other conditions. | Thank you for this thoughtful comment. We agree that there is a large overlap between the two condition groups (concordant and discordant conditions). We have added the following text to highlight this limitation of our paper (page 32 lines 471-476), where in cases where a person lives with both concordant and discordant conditions, we did not explore the individual effects of the two condition groups on HbA1c and mortality. We also did not explore the overlap of the effects of individual conditions within the same condition group, for example, the overlap between chronic liver diseases and alcohol problems.  “There was a large overlap between the concordant and discordant condition groups, so a limitation of our study was that in cases where a person lives with both concordant and discordant conditions, we did not explore this overlap and the individual effects of the two condition groups on HbA1c and mortality. Furthermore, we also did not explore the overlap of the effects of individual conditions within the same condition group, for example, the overlap between chronic liver diseases and alcohol problems.”  However, in responding to your following comment (Reviewer 2 Comment 5 below) we explored the associations between top combinations of conditions and mortality. It is evident in the Taiwan NDCMP (Fig 4b) that a combination of alcohol problems and chronic liver disease had the fourth highest risk for mortality with HR (95% CI) of 3.52 (2.97, 4.18) p<0.001. Therefore, we have shown in Fig3 effect of individual conditions including alcohol problems and chronic liver disease, and now in Fig4 we showed the combined effect of the two on mortality. |
| 5 | The authors mentioned the importance to look at the effect of multimorbidity patterns in patients with diabetes. Considering the big sample size, can the author explore which pattern is more harmful in patients with diabetes, but not only analysing the counts of conditions, which would be very interesting for clinical practice and disease control and prevention. | We have added the following texts to the methods (page 9 lines 241-243), results (page 26), discussion (page 29 lines 365-370, pages 31-32 lines 405-421, page 31 lines 450-451) and conclusion (pages 32-33 lines 483-490) sections of our paper. We have also added two additional forest plots (Fig4a and b) to show the associations between combinations of conditions and mortality.  (page 9 lines 241-243)  “Multivariable Cox proportional hazards models were fitted to all possible combinations of two conditions in addition to T2D to examine their association with all-cause mortality. We present the top 20 combinations in terms of hazard ratios.”  (page 26)  “Fig 4 compares the adjusted HRs of the presence of top 20 combinations (by effect size) of two conditions and all-cause mortality in the UK Biobank (Fig 4a) and the Taiwan NDCMP (Fig 4b). Cardiovascular diseases are present in 18 of the top 20 combinations on mortality in the UK Biobank. Whereas in the Taiwan NDCMP cardiovascular diseases are present in 12 of the top 20 combinations on mortality. In the UK Biobank, a combination of coronary heart disease (CHD) and HF had the largest effect size on mortality with HR (95%CI) of 4.37 (3.59, 5.32) p<0.001. Following the combination of CHD and HF, combinations of CHD and CKD, and HF and dyspepsia, both combinations are associated with greater than four-fold the risk of mortality in the UK Biobank. The remaining top 20 combinations of conditions in the UK Biobank had greater than two-and-a-half-fold risks of mortality. In the Taiwan NDCMP, a combination of painful conditions and alcohol problems had the largest effect size on mortality with HR (95%CI) of 4.02 (3.08, 5.23) p<0.001. Following that, combinations of dyspepsia and alcohol problems, cancer and chronic liver disease, alcohol problems and chronic liver disease, and heart failure and asthma are associated with greater than three-fold the risk of mortality. The remaining top 20 combinations of conditions in the Taiwan NDCMP had greater than two-fold risks of mortality.”  (page 29 lines 365-370)  “Our findings also show that cardiovascular diseases play a significant role in the increased mortality seen in those with multimorbidity, as such conditions were in 18 of the top 20 combinations (by effect size) of two conditions in UK Biobank, while they were present in 12 of the top 20 combinations in the Taiwan NDCMP. We have also shown there are key differences in the top combinations (by effect size) of two conditions in addition to T2D associated with increased risk of death between the two cohorts.”  (pages 30-31 lines 413-429)  “We also contribute to the understanding of patterns of multimorbidity that are associated with poorer outcomes in different ethnic groups through exploring associations between the top combinations of conditions and mortality in a predominantly white population (UK Biobank) and predominantly ethnic Chinese one (Taiwan NDCMP cohort). Our findings show that cardiovascular diseases are present in the majority of the top combinations that are associated with the highest risk of death in both cohorts, consistent with previous literature [23, 24]. Importantly, while we have noted the significant contributions of cardiovascular diseases to increased mortality particularly in the UK Biobank population, our findings also suggest that certain combinations of discordant conditions are also strongly associated with increased mortality. This was particularly marked in the Taiwan NDCMP cohort where alcohol problems, chronic liver disease, cancer, painful conditions and dyspepsia were in the top 4 combinations (by effect size) of two conditions associated with increased risk of death, while only dyspepsia featured as part of a combination in the top 5 combinations in UK Biobank. This highlights the need for further research to consider the importance of ethnic differences when considering the implications of multimorbidity in people with T2D. It also underscores the importance of personalised care, that takes account of individual characteristics including ethnicity, along with number and type of conditions, when managing those with T2D living with multimorbidity.”  (page 31 lines 450-451)  “Our study is also novel in that we explored combinations of conditions that were associated with the highest risk of death.  (pages 32-33 lines 483-490)  “A key finding is that the combinations of conditions with the greatest association with mortality differed between UK Biobank, a predominantly Caucasian population and the Taiwan NDCMP, a predominantly ethnic Chinese population. These findings suggest we need to know more about the influence of different patterns of multimorbidity on outcomes across different ethnic groups in T2D populations. |
| 6 | Lines 212-214, these results (For a given count of concordant conditions, mortality was higher (or survival lower) than for an equivalent count of discordant conditions or any conditions) were not from the same model, can we compare them in such way? | Thank you for this thoughtful comment. We went back to our datasets and checked our concordant and discordant condition count variables and realised that the reference groups of the two variables were inconsistent. We therefore, recoded our concordant and discordant condition count variables so that both reference groups (T2D only) are now exactly the same (ie. the number of deaths/N for each of the reference groups are the same group of study participants, Table 3 and 4). This ensures that we can now compare the results of all of the models. |
| 7 | There is a special issue at the journal, it would be valuable to read these papers: <https://collections.plos.org/cvd-special-issue> | Thank you for this. We went back to read the papers in the PLOS Medicine Special Issue: Cardiovascular diseases, which was very helpful. We have included additional references in our discussion section on page 30 line 419 and page 31 line 439 citing two of the papers from the special issue with particular relevance to our study.  “Our findings show that cardiovascular diseases are present in the majority of the top combinations that are associated with the highest risk of death in both cohorts, consistent with previous literature [24].”  “Therefore it is important to consider the overall multimorbidity disease burden as a way of recalibrating and personalising our clinical focus in managing people with T2D [28].”  24. Tromp J, Tay WT, Ouwerkerk W, Teng T-HK, Yap J, MacDonald MR, et al. Multimorbidity in patients with heart failure from 11 Asian regions: A prospective cohort study using the ASIAN-HF registry. PLoS medicine. 2018;15(3):e1002541.  28. Hoffmann T, Jansen J, Glasziou P. The importance and challenges of shared decision making in older people with multimorbidity. PLoS medicine. 2018;15(3):e1002530. doi: 10.1371/journal.pmed.1002530. |
| ***Reviewer 3***  The strengths of this paper are the use of two very large datasets and a breakdown of different types of multi morbidity. | | |
| 1 | Main suggestion - I suggest the authors discuss more the possible reasons behind the apparently paradoxical association with increased MM and lower HbA1c. For example the authors make little mention of survival bias. One possible reason they see consistent associations between more diseases and lower hba1c is that those with more conditions were more likely have died or be too sick to participate in the studies. They describe the mortality effect nicely but don't appear to make the link. This is important to acknowledge because it means some conclusions need to be reworded eg "these findings suggest a more cautious approach to tight glycaemic control in some MM in type 2 diabetes may be warranted." Although they mention another possibilty - better care, but they also do not explicitly mention possible better and earlier diagnosis - for example people with a heart condition will be tested for HbA1c, and so mild HbA1c is more likely to be picked up as well as treated. | Thank you for this very thoughtful comment. We have added the following text to expand on the possible reasons behind the apparently paradoxical association between increasing multimorbidity count and lower HbA1c in our discussion section (see pages 29-30 lines 383-386).  “This could also mean earlier diagnosis of the range of conditions they are living with. For example, those that have heart diseases could be diagnosed earlier meaning they are more likely to have HbA1c tests that are only mildly elevated, and subsequently receive treatment earlier.”  We have also reworded our conclusion based on your suggestion. (see page 33 line 491) |
| 2 | It would be useful to add non diabetic frequencies of conditions and repeat analyses limited to conditions significant (at p<0.05/42 conditions ) more freq in diabetes. This will help the reader assess whether or not some conditions are prsent in people with diabetes by chance, at similar frequuences as the general population. | Thank you for this helpful comment. It would be interesting to explore the frequencies of conditions in people without diabetes, however this is only possible in the UK Biobank but not the Taiwan NDCMP as it only includes people with diabetes and does not have data on the general population. Therefore we are unable to repeat our analyses limiting to conditions that are more frequent in people with diabetes. This also ensures our analyses are consistent between the two datasets throughout our manuscript. |
| 3 | the tables dont include numbers of deaths. These Ns would be useful to add. | Thanks for pointing this out. We have added number of deaths/N to every row of Tables 3 and 4. See pages 14 and 20. |
| 4 | consider using the new HbA1c scale. | Thank you for the comment. We have considered using the new HbA1c scale, however we have made the decision to still present HbA1c as a percentage. Currently, the World Health Organization (WHO) still supports the use of HbA1c as a percentage and recommends a HbA1c of >6.5% as the diagnostic criteria for diabetes (1). Furthermore, the majority of clinicians are still more familiar with HbA1c presented by a percentage.  This study is one of five studies that form the corresponding author Jason Chiang’s PhD, being completed at the University of Melbourne in Australia. HbA1c has been presented as a percentage across all five studies to allow for comparability of the findings.  1. - World Health Organization. Use of Glycated Haemoglobin (HbA1c) in the Diagnosis of Diabetes Mellitus. Abbreviated Report of a WHO Consultation. WHO/NMH/CHP/CPM/11.1 Geneva, World Health Organization, 2011 |
| 5 | Take care with causal language- statements such as "have effects on mortality" should be reworded as "associated with mortality" | Thank you for the suggestion. We have reworded all causal language statements throughout the manuscript. |
| 6 | Take care with multivariable vs multivariate (sensitivity analyses in methods) | Thank you for pointing this out. We have corrected multivariate to multivariable in the sensitivity analyses section in the methods. (See page 9 line 250) |
